# Characterization and Initial Application of Endophytic *Bacillus safensis* Strain ZY16 for Improving Phytoremediation of Oil-Contaminated Saline Soils

**DOI:** 10.3389/fmicb.2019.00991

**Published:** 2019-05-07

**Authors:** Tao Wu, Jie Xu, Jian Liu, Wei-Hua Guo, Xiao-Bin Li, Jiang-Bao Xia, Wen-Jun Xie, Zhi-Gang Yao, Yu-Miao Zhang, Ren-Qing Wang

**Affiliations:** ^1^Shandong Provincial Engineering and Technology Research Center for Wild Plant Resources Development and Application of Yellow River Delta, College of Biological and Environmental Engineering, Binzhou University, Binzhou, China; ^2^Shandong Key Laboratory of Eco-Environmental Science for the Yellow River Delta, Binzhou University, Binzhou, China; ^3^Institute of Ecology and Biodiversity, School of Life Sciences, Shandong University, Qingdao, China; ^4^Department of Bioengineering, Binzhou Vocational College, Binzhou, China; ^5^State Key Laboratory of Microbial Metabolism, School of Life Sciences and Biotechnology, Shanghai Jiao Tong University, Shanghai, China

**Keywords:** endophytic bacterium, *Bacillus safensis*, hydrocarbon degradation, biosurfactant synthesis, plant growth promotion, salt tolerance

## Abstract

Hydrocarbon-degrading and plant-growth-promoting bacterial endophytes have proven useful for facilitating the phytoremediation of petroleum-contaminated soils with high salinity. In this study, we identified *Bacillus safensis* strain ZY16 as an endophytic bacterium that can degrade hydrocarbons, produce biosurfactants, tolerate salt, and promote plant growth. The strain was isolated from the root of *Chloris virgata* Sw., a halotolerant plant collected from the Yellow River Delta. ZY16 survived in Luria-Bertani (LB) broth with 0–16% (w/v) sodium chloride (NaCl) and grew well in LB broth supplemented with 0–8% NaCl, indicating its high salt tolerance. The endophytic strain ZY16 effectively degraded C_12_–C_32_ *n*-alkanes of diesel oil effectively, as well as common polycyclic aromatic hydrocarbons under hypersaline conditions. For example, in mineral salts (MS) liquid medium supplemented with 6% NaCl, ZY16 degraded *n*-undecane, *n*-hexadecane, *n*-octacosane, naphthalene, phenanthrene, and pyrene, with degradation percentages of 94.5, 98.2, 64.8, 72.1, 59.4, and 27.6%, respectively. In addition, ZY16 produced biosurfactant, as confirmed by the oil spreading technique, surface tension detection, and emulsification of para-xylene and paraffin. The biosurfactant production ability of ZY16 under hypersaline conditions was also determined. Moreover, ZY16 showed plant-growth-promoting attributes, such as siderophore and indole-3-acetic acid production, as well as phosphate solubilization. To assess the enhanced phytoremediation of saline soils polluted by hydrocarbons and the plant-growth-promotion ability of ZY16, a pot trial with and without inoculation of the endophyte was designed and performed. Inoculated and non-inoculated plantlets of *C. virgata* Sw. were grown in oil-polluted saline soil, with oil and salt contents of 10462 mg/kg and 0.51%, respectively. After 120 days of growth, significant enhancement of both the aerial and underground biomass of ZY16-inoculated plants was observed. The soil total petroleum hydrocarbon degradation percentage (a metric of phytoremediation) after incubation with ZY16 was 63.2%, representing an elevation of 25.7% over phytoremediation without ZY16 inoculation. Our study should promote the application of endophytic *B. safensis* ZY16 in phytoremediation by extending our understanding of the mutualistic interactions between endophytes and their host plants.

## Introduction

A large number of oilfields worldwide are situated in areas with high soil salinity (Aizenshtat et al., 1999); therefore, saline and hypersaline soils are frequently contaminated with petroleum because of industrial activities (Fathepure, 2014). Soil polluted by petroleum hydrocarbons poses a serious threat to human health and ecological security, representing a serious environmental problem (Shi et al., 2015). Soil salinization significantly increases the durability of hydrocarbon pollutants and thus increases potential environmental risks resulting from petroleum contamination under natural conditions (Li and Boufadel, 2010). Therefore, an efficient remediation technique for petroleum-contaminated saline soils is urgently needed.

Phytoremediation, which utilizes plants to absorb, concentrate, and degrade organic contaminants from soils, is regarded as a particularly promising bioremediation strategy (Truu et al., 2015) because of its low cost and high sustainability (Chen et al., 2015). Due to the inhibition of plant growth by high salinity, the effects of plants in remediating petroleum-contaminated saline soils have been greatly reduced. To overcome this difficulty, halophytic plants represent candidates for the phytoremediation of petroleum-polluted saline soils (Manousaki and Kalogerakis, 2011). Studies have shown that the growth of halophytes can increase the petroleum pollutant degradation percentage in saline-alkaline soils (Wang et al., 2011). Currently, based on the mutualistic interactions between plants and their endophytes, a combination of plants and endophytic bacteria has been proposed to improve the efficiency of remediation of soil polluted by organic pollutants (Zhu et al., 2014; Arslan et al., 2017). Endophytic bacteria, which colonize various tissues and organs within healthy plants, are usually isolated from surface-sterilized plant tissues (Reinhold-Hurek and Hurek, 2011). Endophytes residing in the internal tissues of plants are exposed to less competition for space and nutrients (Doty, 2008). In the plant-endophyte-based remediation system, host plants supply nutrients and residency to their endophytes (Arslan et al., 2017). Meanwhile, the endophytes promote the growth of the host plant through the degradation of hydrocarbons (Tara et al., 2014). Furthermore, they improve plant growth through various innate mechanisms (Pawlik et al., 2017).

However, until now, very few studies of plant-endophyte-based remediation systems have been reported in the research field of remediation of petroleum-polluted saline soils. Isolation and selection of hydrocarbon-degrading endophytes from halophytes in oil-contaminated sites are particularly critical for exploitation of halophyte-endophyte systems. Here, the hydrocarbon-degrading, salt-tolerant, biosurfactant-secreting, and plant-growth-promoting endophytic *Bacillus safensis* ZY16 was first isolated from the root of a *Chloris virgata* Sw. growing in oil-contaminated saline soil in the Yellow River Delta (Shandong, China).

## Materials and Methods

### Sampling and Isolation

Plant samples of *C. virgata* were sampled from the area of Shengli Oilfield, which is situated in the Yellow River Delta region (N 37°47′05.9″, E 118°39′28.3″). This area is characterized by saline-alkaline soil (Gao et al., 2015), and the sampled halobiotic *C. virgata* grow in saline soil contaminated with crude oil.

The washed plants were divided into three parts: roots, stems, and leaves. The plants parts were sterilized and treated as described previously (Wu et al., 2018), and then the treated plants tissues were used for screening of endophytic bacteria.

### Morphological, Physiological, and Biochemical Characterization

The morphological, physiological, and biochemical characteristics of the selected effective hydrocarbon-degrading endophytic bacterium were analyzed essentially as described previously (Ewa et al., 2013). After incubation on LB agar medium for 48 h (30°C), the colony morphology of strain ZY16 was observed using a stereoscopic microscope system (Motic SMZ-168). In addition, we also observed the cell morphology of strain ZY16 using phase contrast (Eclipse E600) and electron microscopy (JEM-2100). Salt tolerance was assayed in salt-free Luria-Bertani (LB) broth supplemented with 0, 2, 4, 6, 8, 10, 12, 14, 16, 18, and 20% (w/v) sodium chloride (NaCl), and cultures were monitored for growth for 5 days at 30°C. Optimum and limiting temperatures were determined by incubating the isolate at temperatures ranging from 4 to 55°C in LB broth. Growth was scored as positive if the optical density at 600 nm (OD_600_) was higher than 0.5.

### 16S Ribosomal RNA (16S rRNA) Gene Sequencing and Phylogenetic Analysis

Taxonomic classification of the selected effective hydrocarbon-degrading bacterium was carried out using 16S rRNA gene sequence analysis as described previously (Sambrook and Russell, 2001). The 16S rRNA genes of the endophytes were amplified with primers 27F (5′-AGAGTTTGATCCTGGCTCAG-3′) and 1492R (5′-CGGTTACCTTGTTACGACTT-3′) and then sequenced by Sangon Biotech, Co., Ltd. (Shanghai, China). Homologous sequences of the rRNA genes of effective hydrocarbon-degrading strains were identified using the BLAST algorithm homology searching function in GenBank. Multiple sequence alignment was carried out using the software ClustalX 1.81 (Tara et al., 2014). The phylogenetic tree was built via the neighbor-joining method using MEGA 6 (Tara et al., 2014).

### Evaluation of Ability to Degrade Diesel Oil

The capacity of endophytes to degrade diesel oil was evaluated as described previously (Wu et al., 2018). A bacterial suspension (∼1 × 10^8^ CFU/mL) of endophyte was inoculated at a 5% inoculum size into MS medium containing diesel oil (10 g/L) and incubated for 7 days (30°C, 200 rpm). The control experiment involved inoculating with boiled cells of the endophyte. After incubation, the residual diesel oil was extracted with *n*-hexane. The total petroleum hydrocarbons (TPHs) were quantified using a gas chromatograph with flame-ionization detection (GC-FID). The degradation percentages of TPHs were calculated according to the formula proposed by Wu et al. (2018).

### Biodegradation of Long-Chain *n*-Alkanes and Polycyclic Aromatic Hydrocarbons (PAHs)

The ability of the endophytic strain to degrade long-chain *n*-alkanes (e.g., *n*-undecane, *n*-hexadecane, and *n*-octacosane) and common polycyclic aromatic hydrocarbons (PAHs) (e.g., naphthalene, phenanthrene, and pyrene) was investigated using the method described by Zhang et al. (2010). Cell suspensions (MS liquid medium, approximately 1 × 10^8^ CFU/mL) of the tested endophyte were inoculated at a 5% inoculum volume into MS liquid medium supplemented with *n*-undecane, *n*-hexadecane, *n*-octacosane, naphthalene, phenanthrene, or pyrene. The final concentration of each long-chain *n*-alkane or PAH was 200 mg/L. To explore the capacity of the endophyte to degrade these hydrocarbons under hypersaline conditions, three different concentrations of NaCl (2, 6, or 10%) were added to the MS medium for each tested hydrocarbon. The cultures were incubated for 10 days in a shaker at 200 rpm (30°C). The residual hydrocarbons were all extracted with dichloromethane and analyzed using gas chromatography. Differences between treatments with different NaCl concentrations were tested by ANOVA followed by a Tukey’s test via the software SAS version 9.1.3.

### Detection of Biosurfactant Synthesis

Biosurfactant synthesis was detected using the oil-spreading method (Anandaraj and Thivakaran, 2010), liquid surface tension measurements, and emulsification of various hydrophobic substrates (Dastgheib et al., 2008). The screened strain above was cultured in LB medium. LB medium without inoculation, prepared using the same procedure, was used as the control sample. The filtrated supernatant was used to determine the diameter of oil spreading and to measure the surface tension using a K100 tensiometer (Kruss GmbH) via the ring method (Górna et al., 2011).

The ability of the biosurfactant(s) produced by the tested strain to emulsify para-xylene and liquid paraffin was evaluated. In brief, 3 mL of cell-free supernatant was overlaid by 3 mL para-xylene or liquid paraffin and then vortexed for 2 min. The emulsion stability was measured after 24 h, with the emulsification index (EI_24_) calculated using the following equation:

EI(%)24=H/ELH×TS 100

where, *H*_EL_ refers to the height of the emulsion layer, and *H*_TS_ refers to the height of the total solution.

### Detection of Plant-Growth-Stimulating Attributes

Endophytic strains were assayed for the synthesis of siderophores on chrome azurol S (CAS) agar plates, as described previously (Machuca and Milagres, 2003). The prepared CAS agar plates were first divided into equal sectors and then spot-inoculated with the test strain, followed by incubation for 48 h (30°C). Development of a yellow–orange halo surrounding the bacterial growth was regarded as positive for the synthesis of siderophore. Endophytes capable of siderophore production were screened by comparing the diameters of the yellow–orange halos.

Indole-3-acetic acid (IAA) production by the bacterial endophyte was measured via a microplate method, as described by Sarwar and Kremer (1995). Briefly, culture filtrate (150 μL) was dispensed into the wells of a 96-well microplate, followed by the addition of Salkowski reagent (100 μL). The mixture was allowed to react for 30 min, and the color intensity was measured using a microplate reader at 530 nm. The amount of produced IAA was quantified according to a standard curve established with known concentrations of IAA.

The 1-aminocyclopropane-1-carboxylic (ACC) deaminase activity in the endophyte was determined based on its capability to utilize ACC as the sole nitrogen source. The ACC deaminase activity was screened using nitrogen-free DF salts minimal agar medium (Penrose and Glick, 2003). The agar plates contained either (NH_4_)_2_SO_4_ (2 g/L) or ACC (3 mM) as the sole nitrogen source and were incubated aerobically.

The phosphate-solubilizing activity of the endophytic strain was measured in Pikovskays’s medium (Zaidi et al., 2006) supplemented with tricalcium phosphate. Simply, 100 mL Pikovskays’s medium was inoculated with 200 μL culture (∼1 × 10^8^ CFU/mL) of the strain and incubated for 5 days (30°C, 200 rpm). Solubilized phosphate in the cell-free supernatant was quantified by the MO-blue method (Watanabe and Olsen, 1965).

### Effect of Salinity on Biosurfactant Production

Based on the previous salt tolerance assay, the strain was inoculated in salt-free LB broth supplemented with 0, 4, 8, or 12% NaCl. The cultures were monitored for growth (OD_600_) and surface tension at set intervals after incubation at 30°C and 200 rpm.

### Pot Trial Experiment for Endophytic *B. safensis* ZY16 in *C. virgata* Sw.

A pot trial assay (with and without *B. safensis* ZY16 inoculation) was designed, with six replicates for each condition. Each pot contained four plantlets (*C. virgata* Sw.). The oil-contaminated salinized soil applied in this pot trial assay was prepared by the addition of crude oil and NaCl, with final TPH and salt contents of 10462 mg/kg and 0.51%, respectively. Colonization of the tested endophyte in the plantlets was assessed as described previously (Wu et al., 2018). After 120 days, the plants were harvested, and plant biomasses (dry weights) from two treatments were measured. In addition, petroleum residues were extracted from soils using dichloromethane, and the residual petroleum was measured gravimetrically. Student’s *t*-test was applied to compare the means of the two treatments at a 0.01 significance level.

## Results

### Isolation and Characterization of Endophytic Strain ZY16

A total of 23 strains of endophytic bacteria were isolated from the roots, leaves, and stems of *C. virgata* Sw., which grew on petroleum-contaminated saline soil of the Yellow River Delta. Strain ZY16, which was isolated from the root of *C. virgata* Sw., was selected for further study owing to its excellent hydrocarbon degradation, salt tolerance, biosurfactant production, and plant-growth-promoting attributes.

**FIGURE 1:**
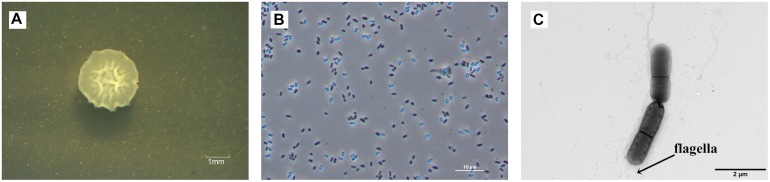
Colony and cell morphology of strain ZY16 observed using phase contrast and transmission electron microscopy (TEM). **(A)** An irregular-edged colony of strain ZY16 on LB agar medium. **(B)** Cell morphology of strain ZY16 using phase contrast microscopy. **(C)** TEM image of rod-shaped endophytic strain ZY16 with polar flagella.

**FIGURE 2:**
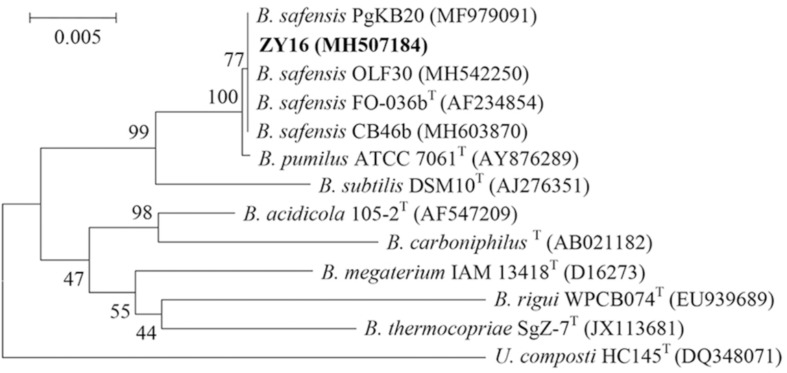
Phylogenetic analysis based on the 16S rRNA gene sequences of *Bacillus safensis* ZY16 and its homologous sequences. *B. safensis* ZY16 is highlighted in bold. Numbers at nodes indicate percentages of occurrence in 1000 bootstrapped trees.

The raised, irregular-edged colonies of strain ZY16 were beige, opaque, and nearly circular (Figure 1A). In addition, under phase contrast and electron microscopy, we observed spore-forming rod-shaped cells with polar flagella that were about 1.1–2.3 μm in length and about 0.6–0.9 μm in width (Figures 1B,C). Physiological and biochemical analyses are shown in Supplementary Table S1. The growth temperature range of strain ZY16 was 20–45°C. Taxonomic classification of strain ZY16 was performed using 16S rDNA sequence analysis. The 16S rDNA sequence of ZY16 was deposited in the GenBank nucleotide sequence data library under accession number MH507184. A phylogenetic tree was built (Figure 2) based on the 16S rDNA sequences via the neighbor-joining method. The results of the BLAST analysis of 16S rDNA sequences indicated that isolate ZY16 is closely related to *B. safensis* PgKB20 (MF979091) and *B. safensis* OLF30 (MH542250). Based on their 16S rDNA sequences and the phylogenetic positions, as well as morphological, physiological, and biochemical characteristics, the isolate ZY16 was designated as *B. safensis*.

### Salt Tolerance and Hydrocarbon-Degrading Ability of Endophytic Strain ZY16

We found that *B. safensis* ZY16 had a high salt tolerance, able to tolerate NaCl concentrations in LB broth of 0–16%. Strain ZY16 grew well in LB broth supplemented with 0–8% NaCl.

**FIGURE 3:**
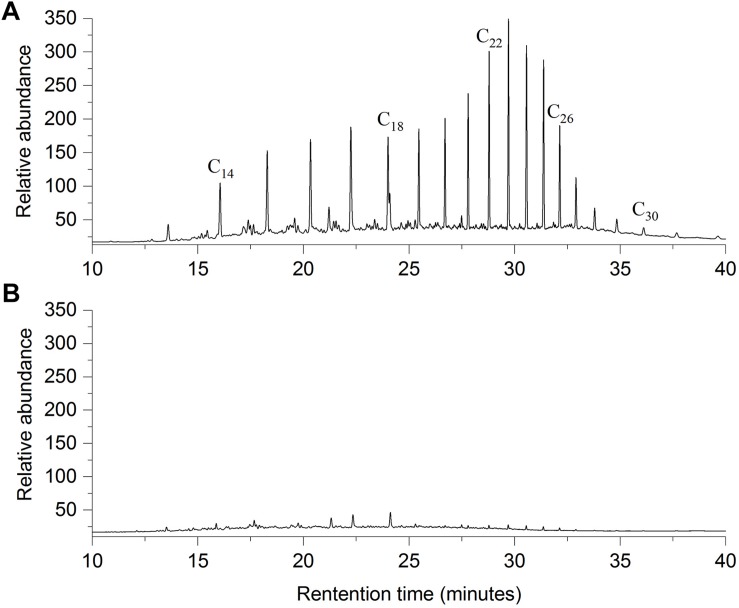
Comparison of GC-FID chromatograms of residual diesel oil between **(A)** control and **(B)** experimental flasks in the biodegradation assay with endophytic *B. safensis* ZY16. The control was inoculated with boiled cells of the isolate. The horizontal axis shows the retention time, and the vertical axis is the relative abundance.

**FIGURE 4:**
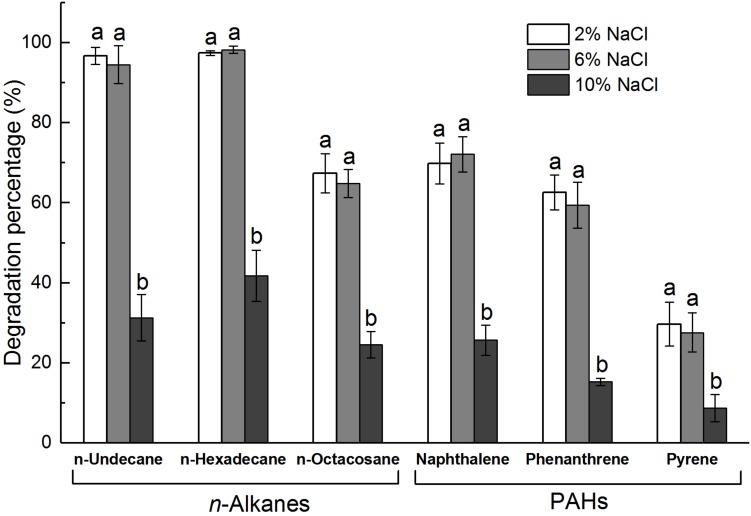
Degradation percentages of hydrocarbons by strain ZY16 at different concentrations of NaCl. Different letters in the same ring compounds represent significant differences in ZY16 degradation percentage under different NaCl concentrations (*P* < 0.05), Tukey’s test. Values are means ± SD (*n* = 3).

*Bacillus safensis* ZY16 also degraded TPHs of diesel oil effectively, with a degradation percentage of 82.5% after 7 days of incubation in MS liquid medium containing 10 g/L diesel oil. By comparing the area peaks of individual *n*-alkanes from the gas chromatograms (Figure 3), we found that strain ZY16 could degrade the C_12_–C_32_ *n*-alkanes of diesel oil. In addition, *B. safensis* ZY16 had a strong ability to degrade long-chain alkanes (*n*-undecane, *n*-hexadecane, and *n*-octacosane) and common PAHs (naphthalene, phenanthrene, and pyrene), even under hypersaline conditions (Figure 4). Compared with those in 2% NaCl, the degradation percentages of all tested long-chain alkanes and PAHs in 6% NaCl did not vary significantly (*P* > 0.05). After 10 days of incubation in MS medium supplemented with 6% NaCl, *B. safensis* ZY16 degraded *n*-undecane, *n*-hexadecane, *n*-octacosane, naphthalene, phenanthrene, and pyrene, with degradation percentages of 94.5, 98.2, 64.8, 72.1, 59.4, and 27.6%, respectively.

### Biosurfactant Production of Endophytic Strain ZY16

**FIGURE 5:**
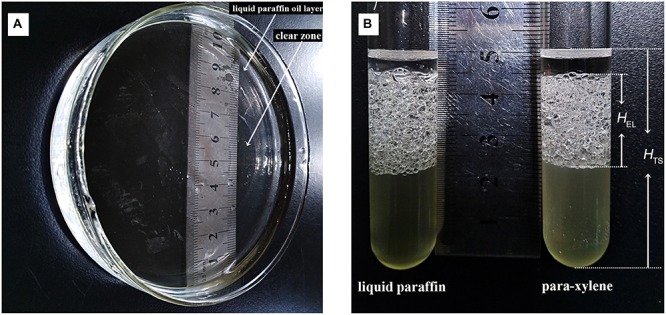
Production of biosurfactant by *B. safensis* ZY16. **(A)** Oil displacement induced by the fermentation broth of a 48-h culture of strain ZY16 was measured using the oil spreading technique. **(B)** Emulsion layers formed by inocula of *B. safensis* ZY16 emulsifying para-xylene and liquid paraffin.

*Bacillus safensis* ZY16 produced biosurfactant, which was confirmed by oil spreading assay, the emulsification index (EI_24_), and surface tension measurements of inocula. In the oil-spreading assay with filtrated supernatant of ZY16, the diameters of the clearing zones on the oil surface were measured up to ∼50 mm (Figure 5A). The values of EI_24_ for strain ZY16 to emulsify para-xylene and liquid paraffin were 41.7–45.3 and 46.6–52.5%, respectively (Figure 5B). No clearing zone or emulsifying activity was observed in the presence of medium without inocula. *B. safensis* ZY16 inoculation decreased the surface tension of the cell-free supernatant from 56.3 to 27.3 mN/m.

**FIGURE 6:**
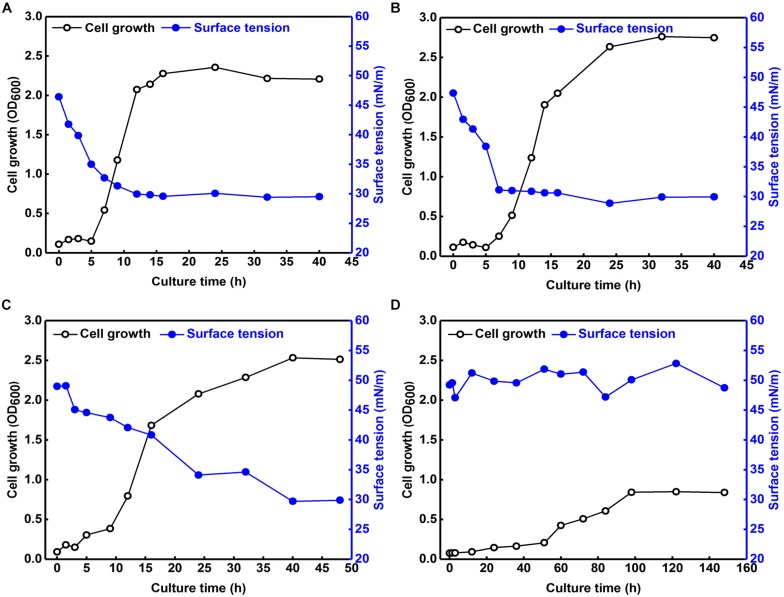
Effects of various NaCl concentrations on the growth of *B. safensis* ZY16 and its production of biosurfactant. **(A)** LB broth without additional NaCl; **(B)** LB broth containing 4% NaCl; **(C)** LB broth containing 8% NaCl; **(D)** LB broth containing 12% NaCl.

We also explored the effects of various NaCl concentrations on the growth of *B. safensis* ZY16 in LB broth and its production of biosurfactant (Figure 6). Strain ZY16 produced biosurfactant extensively in its logarithmic growth stage, with a correlation between biosurfactant production and cell growth. We also found that 12% NaCl inhibited the production of *B. safensis* ZY16.

### Plant-Growth-Stimulating Attributes of *B. safensis* ZY16

We found that *B. safensis* ZY16 has plant-growth-promoting attributes, as well as siderophore and IAA production ability and phosphate-solubilizing activity. We measured the diameters of the orange halo zones in CAS medium at 48 h post-inoculation to quantify the yield of siderophore. The siderophore zone diameters at 48 h were 3.1–3.5 cm. After 72 h of incubation, the yield of IAA produced by isolate ZY16 was 31.4 ± 4.7 mg/L, as measured by the microplate method. Moreover, compared with that in the non-inoculated control (17.6 ± 3.7 mg/L), a highly significant increase (*P* < 0.01) in soluble phosphate (151.5 ± 6.3 mg/L) released into the medium by strain ZY16 was observed. However, the isolate failed to produce ACC deaminase enzyme.

### Pot Trial Experiment With Inoculation of Strain ZY16 for Phytoremediation of Oil-Contaminated Saline Soil

**FIGURE 7:**
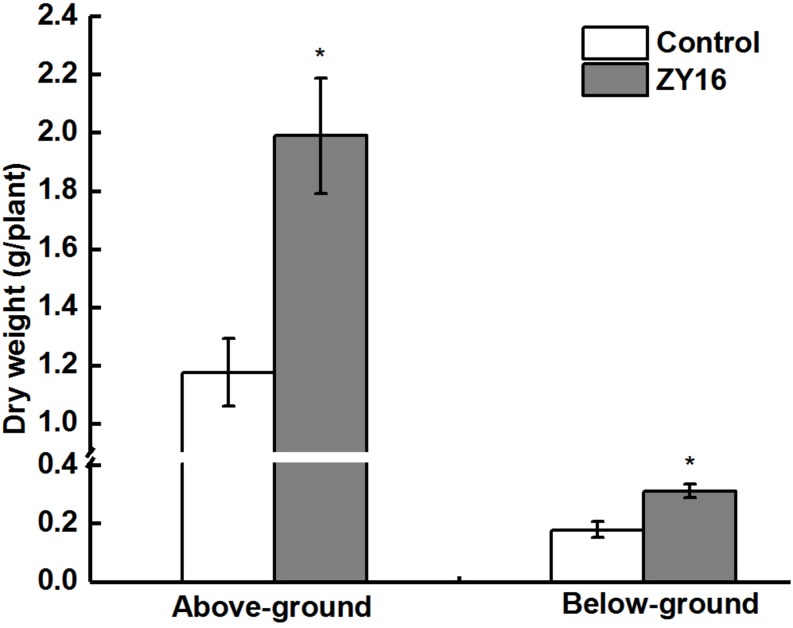
Effect of endophytic isolate *B. safensis* ZY16 on the growth (above- and below-ground biomass) of *Chloris virgata* Sw. Bars indicate standard deviation (*n* = 6). *Indicate statistically significant differences between dried mass of inoculated and non-inoculated plants (Student’s *t*-test; *P* < 0.01).

After planting, inoculation with endophytic *B. safensis* ZY16, and growth for 120 days, the total petroleum hydrocarbon degradation percentage in the soil was 63.2%. Compared with treatment without the inoculation of endophytic strain ZY16, this degradation percentage represented a 25.7% increase, indicating that synergistic activities of strain ZY16 and its host plant *C. virgata* Sw. can effectively promote the degradation of petroleum pollutants in oil-contaminated saline soil. The above- and belowground biomasses (dry weight) of the plants inoculated with endophytic bacteria ZY16 were significantly higher than those not inoculated with ZY16 (*P* < 0.01), with the aboveground biomass increasing by 69.3% and the underground biomass increasing by 75.4% (Figure 7). Endophytic strain ZY16 thus significantly promotes the growth of *C. virgata* Sw., both above and below the ground.

## Discussion

Phytoremediation with plant endophytes represents a powerful approach for improving the bioremediation efficiency of organic pollutants in soils (Feng et al., 2017). Endophytes possessing pollutant-degrading activities and plant-growth-promoting effects are valuable bio-resources for enhancing phytoremediation efficiency. A series of hydrocarbon-degrading and/or plant-growth-promoting endophytic gram-negative [e.g., *Pseudomonas aeruginosa* L10 isolated from *Phragmites australis* (Wu et al., 2018)] and gram-positive bacteria [e.g., *Bacillus* sp. SBER3 isolated from *Populus deltoides* (Bisht et al., 2014)], as well as endophytic fungi [e.g., *Ceratobasidum stevensii* found in *Bischofia polycarpa* (Dai et al., 2010)] have been continuously isolated from their respective host plants. The current study involved the isolation of the endophytic *B. safensis* ZY16 from the root of *C. virgata* Sw. We found that the strain degraded petroleum hydrocarbons, produced biosurfactant, and stimulated plant growth through IAA production, siderophore synthesis, and phosphate solubilizing activity. *B. safensis* ZY16 is a gram-positive bacterium that belongs to the bacterial phylum Firmicutes and the large, pervasive genus *Bacillus* (Satomi et al., 2006). Until recently, reports of endophytic *Bacillus* spp. possessing hydrocarbon-degrading activities and plant-growth-promoting effects have been scarce. Bisht et al. (2014) isolated the endophytic *Bacillus* sp. SBER3, with the activities of PAH degradation and plant growth promotion, from the root of *P. deltoides*. Singh and Padmavathy (2015) isolated an endophytic hydrocarbon-degrading *Bacillus* sp. from *Azadirachta indica*. Strain ZY16 is the first strain of endophytic *B. safensis* isolated from a halophytic *C. virgata* Sw. of the Yellow River Delta that has the characteristics of hydrocarbon degradation, biosurfactant synthesis, plant growth promotion, inorganic phosphate solubilization, and salt tolerance.

Polycyclic aromatic hydrocarbons and alkanes are toxic compounds that are ubiquitous in petroleum oil, posing serious threats to humans and the environment (Bakker et al., 2000). In petroleum-polluted sediments, these toxic compounds can be absorbed and enriched by plants (Meudec et al., 2006). Therefore, endophytes with hydrocarbon-degrading activities in the host plants could directly detoxify these hydrocarbons, enhancing phytoremediation efficiency. This study indicates that the endophytic *B. safensis* ZY16 can degrade *n*-alkanes and PAHs (naphthalene, phenanthrene, and pyrene) effectively. A key limiting factor of hydrocarbon biodegradation is low bioavailability (poor water solubility) of these hydrocarbons. Some hydrocarbon-degrading bacteria can produce biosurfactant, which improves the bioavailability of these hydrocarbons (Kukla et al., 2014). In our study, endophytic *B. safensis* ZY16 exhibited the ability to produce biosurfactant to emulsify various hydrophobic substrates and increase the solubility of hydrocarbons. The halobiotic *C. virgata* Sw. is a dominant plant species in the saline region of Yellow River Delta, China. As a salt-tolerant plant, *C. virgata* Sw. may absorb and accumulate salt, indicating that endophytes in the host plants are exposed to salt stress. In this situation, salt-tolerant endophytes may possess a potential competitive advantage by surviving better than non-salt-tolerant ones. The endophytic *B. safensis* ZY16 in our study was found to exhibit high salt tolerance. Under hypersaline conditions (pure culture), it exhibited a strong ability to degrade long-chain alkanes and common PAHs and to produce biosurfactant. Moreover, in plant-endophyte-based remediation systems, endophytes residing in the internal tissues of host plants often exhibit plant-growth-promoting effects (Pawlik et al., 2017). The endophyte ZY16 was found to have similar plant-growth-stimulating attributes, including siderophore and IAA production and phosphate-solubilizing activity. In summary, the endophytic *B. safensis* ZY16 is a good candidate for the establishment of a plant-endophyte-based remediation system in the field of remediation of petroleum-contaminated saline soils.

Some hydrocarbon-degrading bacteria have shown excellent pollutant-degrading activities under laboratory conditions. However, when they are applied to the removal of petroleum hydrocarbons from contaminated soil under natural conditions, they perform poorly in the bioremediation of organic pollutants because of insufficient nutrition, improper temperature, competition with other strains, *etc.* (David et al., 2009; Nikolopoulou et al., 2013; Kukla et al., 2014). In order to confirm the hydrocarbon-degrading activities and plant-growth-promoting effects of *B. safensis* ZY16 in the phytoremediation of oil-contaminated saline soil, we designed a pot trial experiment. This study demonstrated that *B. safensis* ZY16 was able to increase the biomass of its host plant, *C. virgata* Sw. The association between halophytic *C. virgata* Sw. and *B. safensis* ZY16 exerted a significant effect on the removal of TPHs from crude oil-contaminated soils, compared to that in non-inoculated plants. Our results suggest the immense bioremediation potential of *B. safensis* ZY16 in facilitating phytoremediation for the degradation of hydrocarbons from oil-contaminated soils with high salinity.

## Data Availability

The raw data supporting the conclusions of this manuscript will be made available by the authors, without undue reservation, to any qualified researcher.

## Author Contributions

TW and R-QW designed the experiments. TW, JX, W-JX, and Y-MZ performed all experiments. TW and X-BL analyzed the experimental data and wrote the manuscript. Z-GY, JL, W-HG, and J-BX provided corresponding technical assistance to TW. All the authors have read and approved the final manuscript.

## Conflict of Interest Statement

The authors declare that the research was conducted in the absence of any commercial or financial relationships that could be construed as a potential conflict of interest.
