# Supplementary material for: Characterization and Initial Application of Endophytic Bacillus safensis Strain ZY16 for Improving Phytoremediation of Oil-Contaminated Saline Soils
Source: Front Microbiol. 2019 May 7;10:991. doi: 10.3389/fmicb.2019.00991 (PMC6515983; doi:10.3389/fmicb.2019.00991)
Supplement: Supplementary file 1 [file Table_1.DOCX]

Supplementary Material

Characterization and Application of Endophytic *Bacillus safensis* Strain ZY16 for Improving Phytoremediation of Oil-contaminated Saline Soils

**Table S1 |** Biochemical and physiological characteristics of strain ZY16

| Characteristic | Result | Characteristic | Result | Characteristic | Result | Characteristic | Result |
| --- | --- | --- | --- | --- | --- | --- | --- |
| D-glucose | + | D-galactose | + | D-melezitose | - | Hydrolyses gelatin | + |
| D-mannitol | + | D-fructose | + | D-raffinose | + | ONPG hydrolysis | - |
| Inositol | + | D-mannose | + | Starch | - | Arginine dihydrolase | - |
| D-sorbitol | - | L-sorbose | - | Glycogen | - | Lysine decarboxylase | - |
| L-rhamnose | - | Dulcitol | - | Xylitol | - | Ornithine decarboxylase | - |
| Sucrose | + | Methyl α-D- Mannopyranoside | + | Gentiobiose | + | Citrate utilization | + |
| Melibiose | + | Methyl α-D-Glucopyranoside | + | D-turanose | + | H_2_S production | - |
| Amygdalin | + | *N*-acetylglucosamine | + | D-lyxose | - | Urease | - |
| L-arabinose | + | Arbutin | + | D-tagatose | + | Tryptophan deaminase | + |
| Erythritol | - | Aesculin |  | D-fucose | - | Voges–Proskauer reaction | + |
| D-arabinose | - | Salicin | + | L-fucose | - | NaCl tolerance (%) | 0-16.0 |
| D-ribose | + | D-cellobiose | + | D-arabitol | - | NaCl optimum (%) | 0-8.0 |
| D-xylose | + | D-maltose | + | L-arabitol | - | Growth temperature range (°C) | 20-45 |
| L-xylose | - | D-lactose | - | Potassium gluconate | - | Optimal growth  temperature (°C) | 30-37 |
| D-adonitol | - | D-trehalose | + | Potassium 2-Ketogluconate | - |  |  |
| Methyl β-D- Xylopyranoside | - | Inulin | - | Potassium 5-Ketogluconate | - |  |  |

+ Positive, − Negative
